# The Survey on Near Field Communication

**DOI:** 10.3390/s150613348

**Published:** 2015-06-05

**Authors:** Vedat Coskun, Busra Ozdenizci, Kerem Ok

**Affiliations:** NFC Lab-Istanbul, Department of Information Technologies, ISIK University, Istanbul 34980, Turkey; E-Mails: busraozdenizci@isikun.edu.tr (B.O.); keremok@isikun.edu.tr (K.O.)

**Keywords:** Near Field Communication, NFC survey, Internet of Things, ubiquitous computing, Wireless Body Sensors, NFC ecosystem, NFC security, NFC applications, secure element, NFC usability

## Abstract

Near Field Communication (NFC) is an emerging short-range wireless communication technology that offers great and varied promise in services such as payment, ticketing, gaming, crowd sourcing, voting, navigation, and many others. NFC technology enables the integration of services from a wide range of applications into one single smartphone. NFC technology has emerged recently, and consequently not much academic data are available yet, although the number of academic research studies carried out in the past two years has already surpassed the total number of the prior works combined. This paper presents the concept of NFC technology in a holistic approach from different perspectives, including hardware improvement and optimization, communication essentials and standards, applications, secure elements, privacy and security, usability analysis, and ecosystem and business issues. Further research opportunities in terms of the academic and business points of view are also explored and discussed at the end of each section. This comprehensive survey will be a valuable guide for researchers and academicians, as well as for business in the NFC technology and ecosystem.

## 1. Introduction

Near Field Communication (NFC) is a short range wireless communication technology that emerged only a decade ago, but which has rapidly gained appreciation as a significant contributor to several technologies such as Internet of Things (IoT), Ubiquitous/Pervasive Computing (Ubicomp) and Smart Environment, Ambient Intelligence (AmI), Wireless Sensor Networks (WSN), and Cloud Computing (CC).

NFC fulfills the need to provide secure, short-distance, and implicit paired communication capability in smartphones. The strength of NFC technology arises from its ease of use by triggering the communication just with a simple touch in a short distance, and terminating the communication immediately as the devices detach. One of the most important aspects of NFC technology is its inherent security, since the communication range is extremely short. In NFC communication, bringing two devices very close to each other starts communication and separating the devices beyond a certain limit terminates the communication immediately.

NFC technology invalidates many other devices, components, materials, and items so that only a smartphone used as an integrated everyday life object would be sufficient to realize all daily activities [1,2]. Smartphones could be used to lock/unlock the house, car, and office doors, pay for the newspaper, exchange business cards, pay for public transportation, tip a doorman, help disabled or elderly people cope with their daily life, and much more.

NFC not only brings simplicity to our lives, but also creates additional opportunities for entrepreneurs as well. Mobile Network Operators (MNOs), financial institutions, transportation authorities, device manufacturers, and Service Providers (SPs) are increasingly aware of the NFC technology since its ecosystem creates promising business opportunities. As of today, most smartphones are sold with an integrated NFC hardware module, and almost all smartphone/Mobile Operating Systems (MOSs) have NFC support, which are important evidence of its popularity and usefulness through the dissemination of the technology.

NFC is compatible with the existing infrastructure spawned for Radio Frequency Identification (RFID) technology such as passive RFID tags and contactless ISO 14443 compatible readers. In order to engage in an NFC interaction, a user needs to touch her smartphone alternatively to an NFC tag, another smartphone, or an NFC reader. The smartphone communicates with each mentioned device in a different fashion. When touched to an NFC tag, the smartphone reads/writes data from/to the tag. When touched to another smartphone, they exchange data. When touched to an NFC reader, the reader reads the data stored in the Secure Element (SE) of the smartphone. An operating mode name is given to each interaction: reader/writer mode to the tag interaction, peer-to-peer mode to the smartphone interaction, and card emulation mode to the reader interaction.

The pioneering work on NFC began about a decade ago, as covered by our previous review on the topic [3]. We observed the growing momentum in the technology, including technical aspects, application development efforts, SE alternative proposals, and new ecosystem models, which motivated us to provide an up-to-date study. The main objective of our current study is to provide insights into what has been happening in all aspects of NFC technology and address open research areas for further improvement.

This survey is based on articles in journals and mostly conference proceeding papers. We exclude some writings published as editorials, news reports, book reviews, or white papers. The literature search was based on two descriptors: “NFC” and “Near Field Communication”. The search for papers was conducted using electronic databases such as IEEE/IEE Electronic Library, Association for Computing Machinery, ISI Web of Knowledge, Academic Search Complete, Computer and Applied Science Complete, Science Direct and Emerald Full Text.

Sometimes the abstract, but mostly the full text of each article was read to identify whether the article had high relevance to NFC. The literature review strategy followed for this study was an iterative process, hence, we tried to find and add new studies about NFC to the survey.

In conjunction with the above-mentioned objective, the remainder of this study is organized based on the major research areas of NFC technology: in Section 2, we present the NFC technology, NFC communication, and related studies. In Section 3, SE essentials, SE management issues, and related studies are reviewed. Section 4 contains the applications and services making use of NFC technology. In Section 5 security and privacy issues regarding NFC technology are discussed, and Section 6 provides usability studies on NFC technology. In Section 7 ecosystem and business models for NFC technology are reviewed. Concurrently, we provide useful guidelines and mention open research issues for the reader at the end of each section.

## 2. NFC Technology Essentials

NFC technology is a short-range half duplex communication, which was jointly developed by Philips and Sony in late 2002 for contactless communications. NFC relies on the inductive coupling principle between transmitting and receiving devices, and differs from far field RF communication, which is used for longer-range wireless applications. We provide an account of NFC technology in this section.

### 2.1. NFC as a Contributor Technology

A sensor is a device that aims to detect some event or characteristics of its environment. Analogous to the Internet of computers, sensor technology has the capability to provide a large network of sensors with the ability to obtain data from the environment and process it afterwards [4] in areas including air, ground, water, and even underwater [5,6]. The idea of IoT arose in this way, as being the concept of variety of things, which interact with each other to reach some common goals [7]. Currently such IoT include RFID and NFC tags, barcodes, sensors, actuators, and smartphones, *etc*., which may potentially include additional items in the future.

UbiComp refers to the next level of interaction between human and computers, where computing devices are completely integrated into our everyday life and objects around. UbiComp is a model in which human beings do not design their activities according to the machines, instead, machines change their forms to adjust to human behaviors [8].

AmI also has a crucial role in UbiComp, especially in living environments such as homes. It is defined as an information technology paradigm that makes use of context-sensitive digital environment by being adaptive and responsive to people’s needs, habits, gestures, and emotions. By embedding technology into objects, AmI establishes effortless interaction and enables information to be accessed anywhere and anytime [9]. Ubiquitous communication including NFC technology is also one of the primary technologies that promote AmI [10]. IoT, UbiComp, Smart Environment, and AmI are some conceptual responses those are provided by the scientists in response to the public need to simplify their lives to technology. Herein, NFC technology plays an important role in promoting almost all of those concepts. NFC technology brings ease of use, provides physical object elimination, enables implicit coupling, and much more.

Needless to say, NFC is a major contributor technology for the promotion of IoT and context-aware Smart Environment. The authors of [4] provide a good example that integrates NFC technology into the IoT concept. Each smart classroom is equipped with NFC tags and readers to collect real-time information and to manage these smart classrooms accordingly. Another study [11] defines the education system called NFC Mobiquitous—mobile and ubiquitous—Learning System (MoLS) where course contents are accessible by anyone, anytime, and anywhere with a simple interaction of NFC tags and smartphones.

Another important technology that also promotes IoT is WSNs, which are composed of sensor nodes deployed in an area [5]. WSNs are used in a variety of areas such as forest fire detection, healthcare monitoring, natural disaster prevention, and so on. In addition to using passive components (sensors) in the network, active devices (actuators) are added to perform some action, in which case the network is known as a Wireless Sensor and Actuator Network (WSAN). The gathered data are first transmitted to a base station to transfer it to a final location over the Internet, which enables execution of applications including health care, diagnosis, emergency treatment and so on [12]. Another notable specific form of WSN is the Wireless Body Area Network (WBAN), which is a wireless network of wearable computing components placed on the body to collect data. WBANs have arisen increasingly to meet the needs of growing markets such as health monitoring, emergency assistance, video games, and smart clothes [13]. Another study [14] provides a good example of the benefits of NFC technology in WBANs. The authors introduce a smart mobile system that collects sensor data via Bluetooth, uses NFC as a facilitator for establishing Bluetooth connection, and streams data simultaneously to a central server via the Internet.

NFC technology also provides important contributions to the development of CC services. In particular, storing private data on the Cloud instead of on smartphones is an important recent development; and several significant studies on Cloud-based NFC services exist [15,16,17] which are further discussed in Section 3.

### 2.2. NFC Communication

**Figure 1:**
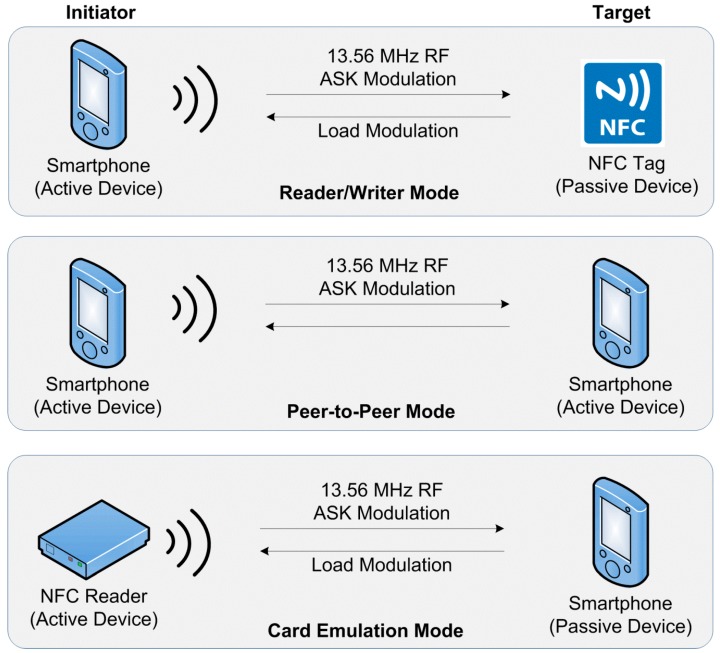
NFC interaction styles and operating modes.

NFC communication occurs between two NFC compatible devices placed within a few centimeters of each other using the 13.56 MHz operating frequency (Figure 1). It provides easy communication between various NFC devices on ISO/EC 18000-3 air interfaces, with transfer rates of 106, 212, and 424 Kbits per second. The device that starts the communication is called the initiator, while the respondent is known as the target. NFC smartphone and NFC readers uses their own power, hence are active devices, whereas an NFC tag uses the power of the other party, and hence is called a passive device. All initiator devices are usually active devices, however a target device can be either active or passive, depending on the operating mode.

Three types of NFC devices are involved in NFC communication: smartphones, NFC tags, and NFC readers. The possible interaction styles among NFC devices provide three different operating modes as shown in Figure 1: reader/writer, peer-to-peer, and card emulation operating modes where communication occurs between a smartphone on one side, and an NFC tag, another smartphone, or an NFC reader on the other side, respectively [8,18].

Each operating mode uses its own communication interfaces and standards; ISO/IEC 18092 NFCIP-1 [19], ISO/IEC 21481 NFCIP-2 [20], JIS X 6319-4/Felica [21] and ISO/IEC 14443 [22] contactless smart card standards (referred to as NFC-A, NFC-B and NFC-F, respectively) in the NFC Forum specifications on RF layers. Moreover, each operating mode has different technical, operational and design requirements (see the next sections).

The NFC protocol occurs using two communication modes: active and passive mode [19]. In active communication mode, both devices use their own energy to generate their RF field to transmit the data. In the passive communication mode, only the initiator generates the RF field while the target device makes use of the energy that is already created.

In NFC communication that occurs from an active device to a passive device, the Amplitude Shift Keying (ASK) modulation technique is used at all possible data rates. In the case of communication from a passive device to an active device, the load modulation technique is used. In terms of coding schemes, it uses Non-Return-to-Zero Level (NRZ-L), Manchester, or Modified Miller coding techniques, which depend on the data rates and standards used on the RF interface (*i.e.*, JIS X 6319-4/Felica and ISO/IEC 14443 contactless smart card standards).

There exist several studies on improving the efficiency of NFC communication through novel modulation techniques. One study [23] deals with the increase of data rates for proximity coupling of NFC devices at 13.56 MHz, and compares the performance of the ASK and PSK modulation schemes in a real environment. It concludes that PSK performs 23% better than ASK in terms of field strength requirements and energy efficiency. Another study [24] focuses on a highly efficient 13.56 MHz NFC transmitter to improve the efficiency of ASK modulation.

Some authors have proposed a modulation technique called Active Load Modulation (ALM) to overcome the limitations of using passive load modulation [25]. Another study [26] handles the load modulation bottlenecks and provides ALM concepts, which enhances the card emulation mode operation.

The authors of [27] focus on high-speed NFC transmissions based on Extended Binary Phase Shift Keying (EBPSK) modulation and they present its advantages over the existing NFC system. The authors of [28] present a multi-level Phase Shift Keying (PSK) modulation to increase the data rate of 13.56 MHz inductively coupled systems. The authors of [29] examine Quadrature Phase Shift Keying (QPSK) in which modulation with additional data transmission is studied to enhance NFC transactions. The authors of [30] propose a Direct Antenna Modulation (DAM) technique to increase the performance of NFC link since most NFC systems operate at low RF frequencies.

### 2.3. NFC Smartphones

**Figure 2:**
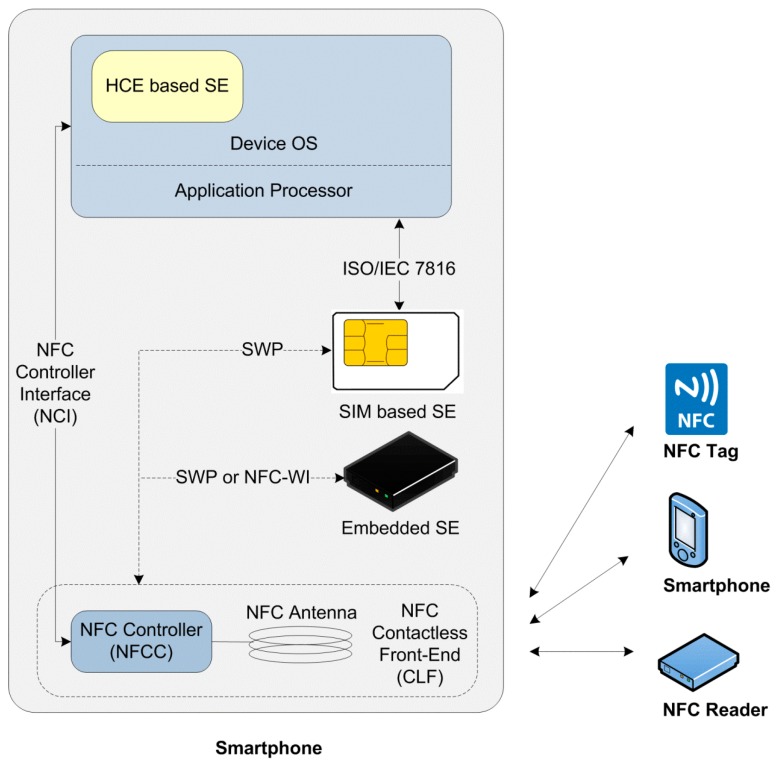
General architecture of a NFC smartphone.

NFC smartphones are the irrevocable component of NFC communication, which is typically composed of various integrated circuits such as the NFC communication module depicted in Figure 2. The NFC communication module is composed of an NFC Contactless Front-end (NFC CLF), an NFC antenna and an integrated chipset referred to as an NFC Controller (NFCC) whose function is to manage the emission and reception of the signals, and modulation/demodulation.

A variety of studies have been performed to improve the NFC module and NFC antenna. Many studies [31,32,33,34,35,36,37,38,39,40] focus on optimization of NFC antenna design for enhancing the performance and operating distance of the NFC antenna, as well as for impedance adjustment issues.

Inkjet printed antennas [41,42] for passive components such as NFC tags are another popular NFC antenna design approach. Antenna design formulas optimizing bandwidth and power transfer efficiency [43], evaluation of eavesdropping range depending on the antenna design [44], development of mutual coupling of NFC antennas and coupling models for system performance estimation [45,46,47], resonant coupling method for enabling high quality NFC system [48,49] have also been studied extensively.

The mutual inductance on NFC antennas is another popular research area, which affects impedance matching and transmission efficiency for NFC applications. Some studies [50,51] provide Equivalent Circuit Analysis for inductively coupled NFC antennas to examine the mutual inductance parameters.

Moreover, novel NFC transceiver and chipset design approaches have been studied [52,53,54,55,56] for improving communication quality and security. Adaptive tuning strategies for NFC transmitter module to compensate receiver influence [57], quadrature carrier cancelling receiver to solve the self-jamming problem and increasing the sensitivity of NFC receivers [58] are other important issues for NFC system design.

Optimization of power requirements and energy saving interface models in NFC chipset design are proposed in studies such as NIZE [59] and PTF Determinatior [60]. Adaptive and dynamic field strength scaling methodologies are studied for energy saving purposes [61,62].

The SE of NFC technology enables secure storage and secured transactions among NFC devices [63]. Currently, the promising SE alternatives for NFC transactions are embedded hardware-based SE, SIM-based SE, and Host Card Emulation (HCE). For the embedded hardware-based SE option, the NFC Controller is connected to the SE through either Single Wire Protocol (SWP) [64] or NFC Wired Interface (NFC-WI, ECMA 373, S2C) [65]. SWP is the most adopted protocol between an NFC Interface and SE, which can be used in all SE form factors, and provides optimal interoperability with several protocols [66].

Another important NFC smartphone component is the NFC Controller Interface (NCI) which is a standard defined by the NFC Forum [67]. NCI is the interface between the NFCC and a device’s main application processor, *i.e.*, its host controller [68]. NCI enables the integration of chipsets manufactured by different chip manufacturers, and defines a common level of functionality and interoperability among the components within an NFC device.

In one study [69], the authors indicate that mobile device manufacturers expect NFC systems to be operating system (OS) independent, hardware independent, and OS application framework adaptive. Actually in today’s smartphones, NFC stacks hardly meet all these requirements due to business model-related issues. In addition, the authors of [69] propose a new NFC stack architecture for mobile devices by analyzing OS services, ETSI standards and NFC Forum standards. The proposed NFC stack runtime environment is also validated on the Android and Windows Phone OSs.

### 2.4. NFC Operating Modes

NFC technology benefits from various elements such as smart cards, smartphones, NFC tags, and card readers. Various standardization bodies define how NFC technology should be integrated into smartphones and other related devices in all layers. The common vision of all NFC standardization bodies includes the ease of access, interoperability, and security.

The most important association that focuses on developing and improving NFC technology is the NFC Forum [67]. It is a non-profit Standard Setting Organization (SSO) that was established with the initial aim of enabling NFC technology, and promoting it globally thereafter. Up to now, NFC Forum has provided diverse specifications such as Logical Link Control Protocol (LLCP) [70], NFC Tag Types [71,72,73,74], NFC Data Exchange Format (NDEF) [75], NFC Record Type Definitions (RTDs) [76,77,78,79,80] and so on. Three NFC communication modes are defined based on the paired NFC devices. The communication protocols and standards are defined as operating mode specific.

#### 2.4.1. Reader/Writer Mode Communication Essentials

**Figure 3:**
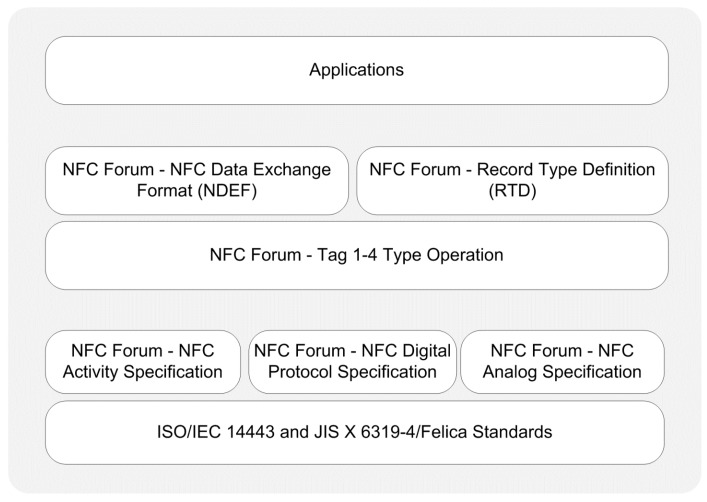
Protocol stack of reader/writer operating mode.

In reader/writer operating mode, a smartphone initiates the communication as an active device, and can both read from and write to an NFC tag. NFC tags are some form of passive RFID tags. Figure 3 provides a protocol stack illustration for reader/writer mode.

*Physical Layer:* The RF layer of the NFC communication is based on the ISO/IEC 14443 and JIS X 6319-4 Felica contactless smart card standards. Depending on these existing standards, the NFC Forum defined additional specifications for the physical layer, which are the analog specification, digital protocol specification, and activity specification. The analog specification is related to the RF characteristics of NFC devices and determines the operating range of devices [81]. The digital protocol specification refers to the digital aspects of ISO/IEC 18092 and ISO/IEC 14443 standards, and defines building blocks of communication [82]. The activity specification defines the required activities which set up communication in an interoperable manner based on the digital protocol specification such as polling cycles or when to perform collision detection [83].

*Data Link Layer:* In this operating mode, a smartphone is capable of reading NFC Forum-mandated tag types, which are Types 1–4, as defined by the Tag 1–4 Type Operation Specifications [71,72,73,74]. These specifications indicate the commands and instructions used by smartphones to operate the NFC Forum mandated tags.

The NFC Forum also defines standardized NDEF, the data exchange format between two NFC devices [75]. The NFC Forum mandated tag-enabled read and write operations on the data link layer by using the NDEF and Record Type Definitions (RTDs) from/to a tag. NDEF has a binary message format that encapsulates one or more application specific payloads into a single message [75]. NDEF is also defined as a standardized format for storing formatted data on NFC tags and for transporting data across a peer-to-peer NFC link [84].

An NDEF message contains one or more NDEF records. Records can be chained together to contain large payloads. A record is the unit for carrying a payload within an NDEF message. Each NDEF record contains values describing its payload, payload length, and payload type. The length of NDEF records is variable, and definitions of NDEF record fields can be found in the specifications [75,76]. The most important one is the Type Name Format (TNF) field, which indicates the structure of the NDEF record. The NFC Forum provides various record types for NDEF messaging format such as smart poster, text, URI, signature [77,78,79,80].

*Application Layer:* Smartphone applications in reader/writer mode may create their own data exchange format, or may also optionally use NDEF. Smart poster applications are examples of NDEF-based applications and handle the tag data based on NDEF specifications. On the contrary, non-NDEF-based vendor-specific smart poster applications are also possible.

#### 2.4.2. Peer-to-Peer Mode Communication Essentials

**Figure 4:**
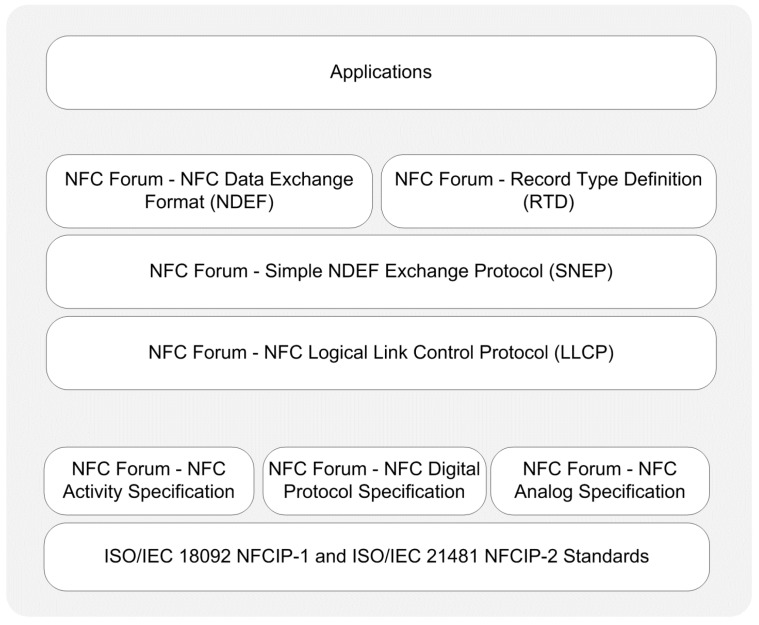
Protocol stack of peer-to-peer operating mode.

In peer-to-peer mode, two smartphones establish a bidirectional connection to exchange data. In this mode, smartphones can exchange any kind of data such as business cards, digital photos, or any data specific to an application. Figure 4 provides a protocol stack illustration for peer-to-peer mode.

*Physical Layer:* The RF interface is standardized by ISO/IEC 18092 as NFCIP-1 and ISO/IEC21481 as NFCIP-2, which enables request-response model between two active devices [32], and also ISO/IEC 21481 as NFCIP-2, which detects and selects the communication protocol that will be used in peer-to-peer communication [20].

According to one study [85], the NFCIP-1 protocol allows error handling, provides an ordered data flow, and performs reliable and error free communication in the link layer. In another study [86], a simulation model for the NFCIP-1 over the network simulator is presented. The study indicates that NFCIP-1 protocol needs to be supported with other techniques such as flow control mechanisms. Another study [87] presents realization of an IP link by tunneling over the NFCIP-1 protocol, which enables devices to exchange data over the network easily. Such a tunneling implementation may bring new possibilities for peer-to-peer mode applications.

In addition to NFCIP-1 and NFCIP-2, the NFC Forum has the defined analog specification, digital protocol specification, and activity specification for the physical layer in peer-to-peer communication [81,82,83].

*Data Link Layer:* To support peer-to-peer communication between two NFC-enabled devices, the NFC Forum has standardized LLCP [70,87]. LLCP provides a solid ground for peer-to-peer mode applications and enhances the basic functionalities provided by the NFCIP-1 protocol. According to the NFC Forum, LLCP provides five important services: connectionless transport, connection-oriented transport, link activation, supervision and deactivation, asynchronous balanced communication, and protocol multiplexing [70].

As mentioned in [88], NFCIP-1 makes use of an initiator-target paradigm that defines the initiator and target devices prior to starting the communication. However, LLCP makes peer-to-peer transactions smoother since the devices are identical in LLCP communication [88]. After the initial handshake, the decision to assign initiator and target functions to each device is made by the application running in the application layer. Another study [89] presents a short comparison between NFCIP-1 and LLCP through a social networking application. Both NFCIP-1 and LLCP are tested during the application development. By using LLCP, users can easily perform peer-to-peer communication; they do not need to agree beforehand about the identity of the initiator and the target.

In some studies [90,91], a secure version of the LLCP is defined as LLCPS (*i.e.*, the Logical Link Control protocol secured by TLS), which protects the transactions in peer-to-peer mode. The protocol is validated by two experimental platforms and provides strong mutual authentication, privacy, as well as integrity.

Another important protocol in this layer is the Simple NDEF Exchange Protocol (SNEP) [92] that allows an application on an NFC-enabled device to exchange NDEF messages with another device in peer-to-peer mode. The protocol makes use of the LLCP connection-oriented transport mode to provide a reliable data exchange [92].

In accordance with peer-to-peer protocols, one study [88] analyzes available peer-to-peer protocols and presents OPEN-NDEF Push Protocol (NPP) as an open source library. NPP is a simple protocol built on top of LLCP, which is designed by Google to push an NDEF message from one device to another on Android devices [88]. In another study [93], the authors present OPEN-SNEP library as an update to NPP, and analyze technical details of the OPEN-SNEP solution. The main differences between NPP and SNEP from a developer point of view are presented with use cases as well.

Similar to the reader/writer mode applications, peer-to-peer mode applications also use the standardized NFC Forum’s NDEF messaging format and RTDs.

*Application Layer:* Peer-to-peer mode applications may optionally run over SNEP or other protocols. Up to now, diverse applications have been performed in peer-to-peer mode due to its highly standardized nature and various use case scenarios such as printing a file from a smartphone, business card exchange, *etc*. It is also possible to see some novel applications that combine NFC and Bluetooth technologies to create secure peer-to-peer applications such as secure money transfer between mobile devices [94].

The NFC Forum also provides Reference Application Specifications to promote NFC-based applications and services such as Personal Health Device Communication [95] for the acquisition of personal health data from personal health devices using NFC and Connection Handover [96] to establish an alternative wireless communication such as Wi-Fi or Bluetooth between two NFC-enabled devices. A variety of applications can be enabled by using the Connection Handover protocol, such as printing to an NFC + Bluetooth printer, or streaming video to an NFC + WLAN television set.

#### 2.4.3. Card Emulation Mode Communication Essentials

**Figure 5:**
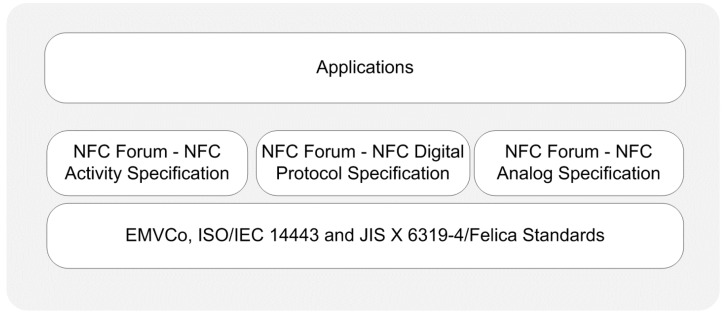
Protocol stack of card emulation operating mode.

In card emulation mode, as the user touches a smartphone to an NFC reader, the smartphone behaves like a standard smart card; thus, the NFC reader interacts with the SE directly. Figure 5 provides a protocol stack illustration for card emulation mode.

*Physical Layer and Data Link Layer:* The RF layer of the NFC communication is based on both ISO/IEC 14443 Contactless Smart Card and JIS X 6319-4 Felica standards. It uses digital protocol and analog techniques similar to smart cards, and it is completely compatible with the smart card standards based on ISO/IEC 14443 Type A, Type B and Felica. Moreover, it uses the NFC Forum’s analog, digital protocol, and activity specifications for the physical layer, which are already mentioned above [81,82,83].

*Application Layer:* Card emulation mode includes proprietary contactless card applications such as payment, ticketing, and access control. These applications are based on the ISO/IEC 14443 Type A, Type B and Felica communication interfaces.

### 2.5. Further Research Opportunities

Technically, peer-to-peer communication can be performed between any two NFC active devices such as smartphones and NFC readers; but up to now only communication between two smartphones has been studied in the literature. Various possibilities for research into NFC technology exist for academicians and researchers. Some of the challenging studies on technological essentials are listed below.

- Development of alternative protocols for NFC additional to SNEP for promoting P2P transactions.
- Extended investigation of LLCP with a simulation model.
- Exploration of low-power modulation schemes.
- Design of new modulation and coding techniques for NFC.
- Analysis of low energy consumption models on NFC readers.
- Development of simulation models for NFC tags as transponders.
- Examination of NFC Dynamic Tags which enable dynamic data, rather than NFC Static Tags.
- Integration of NFC technology with OS and novel NFC Stack Architecture models.
- Development of Quality of Service mechanisms in NFC context.

## 3. Secure Element

As smart cards have been used for storing private information, additional issues including where to save multiple applications and their credentials, how to provide authentication and identification, and how to satisfy PIN management and signatures have arisen. These issues have forced major standard providers to define a new concept known as SE, which is defined as the concept of storing and processing sensitive data on mobile components such as smartcards and smartphones. Creation of an SE requires a secured and controlled environment so that security requirements such as secrecy, authentication, or signature can be satisfied.

### 3.1. SE Essentials and Alternatives

An SE is built into secure crypto chips on the smart card where private data (e.g., credit and debit card numbers, mobile signature credentials, *etc*.) and corresponding smart card applications are saved and kept secure [8].

In most SEs, a Virtual Machine (VM) exists and it is associated with an executable VM called Security Domain (SD). A SD is a protected area on the card, which securely stores cryptographic keys [97]. Traditional SEs comprise CPU for processing, Read Only Memory (ROM) for storing the operating system, Non-Volatile Memory (NVM) for storing applications and related data, and Random Access Memory (RAM) [98]. SEs cannot trigger any application themselves; rather they receive data requests from applications, and return responses. The commands they use are standardized by the ISO7816 standard [15,98].

As both smartphones and cellular communication technology have been improved in recent decades, smart cards have also became a major component of smartphones. Smart cards used in smartphones are known as Universal Integrated Circuit Cards (UICCs), also referred to as Subscriber Identity Module (SIM). Despite the fact that its aim was to identify the phone subscriber correctly, it also includes a SE for additional usage. UICCs are compliant with smart card standards and support the hosting of multiple applications on the same card [99].

SE is an important asset in mobile technology, since new business models and partnerships should be established with the party that owns the SE. Because of the incredible potential for high-value monetary transactions in NFC, the ecosystem actors (*i.e.*, banks, MNOs, or handset manufacturers) have tried to impose a specific model through which they could benefit most. The ad hoc model was to use UICC as the home of SE, which obviously creates a great advantage for the MNO, the manager of the SIM. Other actors in the game did not accept the UICC-based SE ownership model, and the efforts of other actors have initiated the creation of different SE alternatives.

The next option was to use embedded SE, which obviously delighted smartphone manufacturers. This chip is integrated into the smartphone during the manufacturing process and can be personalized after the device is delivered to the end user [99].

Other SE alternatives have also been proposed to enable secure storage and management of private data and card applications, but most of them were abandoned after a while due to inadequate attention and support from stakeholders.

The Secure Digital card was initially promoted in order for the users to increase the storage capacity of smartphones. Lately, it appeared to be a brilliant idea to use Secure Digital cards as the place for facilitating SE. Having this option in smartphones delighted Service Providers since they would neither be dependent on either MNOs (as in the UICC-based SE case) nor the manufacturers (as in the embedded SE case). Nevertheless, this option ended up being a disappointment for several reasons. After the decrease in the costs of embedded storage units of smartphones, there was not much need to provide external storage options such as Secure Digital. Additionally, not all Secure Digital cards, but only the ones that were manufactured accordingly could be used as alternatives to SE, hence the users who wished to use SE had to buy specific Secure Digital card models anyway, and they had to pay more to do so, due to the higher cost of SE-enabled Secure Digital cards. Secure Digital-based SE is no longer an alternative [98].

Software-Based SEs, also known as Trusted Mobile Base (TMB), became interesting for a while. TMB was hosted at the root of the smartphones. It was defined as a secure isolated section on the Core Processor Units of the smartphones [100], but it also lost its popularity.

NFC Stickers with embedded NFC antenna were produced in order to launch pilot studies and deploy NFC services quickly by both providing a SE and providing NFC functionality to non-NFC-enabled smartphones [100]. However the integration of NFC into almost every smartphone diminished its preference.

**Figure 6:**
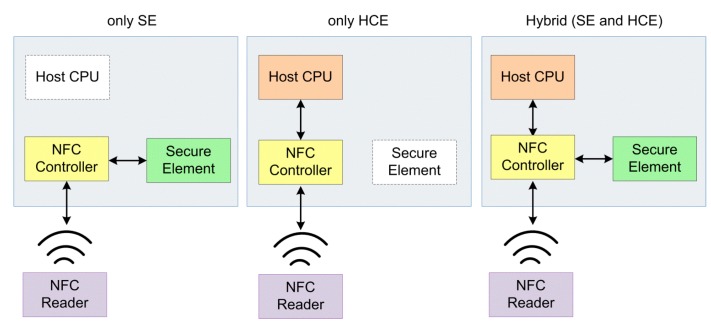
SE and HCE architecture comparison.

Host Card Emulation (HCE) is the latest and most significant approach to store, access and manage private data, which separates the card emulation mode function of the smartphone from SE. The smartphone still performs card emulation mode functions, but the private data is stored in a different location such as on the smartphone or Cloud. HCE has its own protocols and standards to create and manage secured areas (Figure 6).

HCE provides more independence to SPs, but a drawback of Cloud-based HCE case is that poor user experience occurs if network latency exists [101]. Moreover, a smartphone may not connect to the Internet during the transaction for some reason, and thus cannot communicate with the SE on the cloud. In a study that includes a cloud-based HCE approach [16], the data is retrieved from the virtual SE on the cloud to the smartphone’s internal SE in encrypted format. The contactless terminal reads data from SE and performs required operations such as a credit card authorization afterwards. In this model, the internal SE serves as a secure temporary storage.

Since HCE technology and Cloud-based HCE are new concepts, only a few studies for different architectures have been performed. In one of these studies, a remote server of SEs is proposed and named Cloud of Secure Elements (CoSE). In that study; a Smartphone remotely uses a SE that is hosted on the servers by establishing a secure TLS channel, and the system is able to solve trust issues for Internet users, mobile applications, and virtual machines environments [15]. In another proposed solution [16], Cloud-based SE securely stores personal data such as credit or debit card information, user identification number, loyalty program data, payment applications, PINs and personal contacts securely. Furthermore, UICC in the smartphone stores authentication data such as keys, certificates, protocols, and cryptographic mechanisms. In this case, SE in the smartphone is only used for authentication, and the data is stored on the Cloud [16]. As a result, the only remaining promising SEs as of today are UICC-based SE, embedded SE, and HCE.

### 3.2. SE Management

SEs can be remotely managed using Over-the-Air (OTA) technology that enables remote installation of applications, activation and deactivation, remote service management, lifecycle management of applications, and other online services [8]. SEs are managed according to the GlobalPlatform specifications [102]. As a result of these specifications; SD provides storage for applications and cryptographic keys, which are protected by mutual authentication using symmetric keys [98]. In one study [103], a new SE management named Over-the-Internet is proposed which transmits the SE management commands over a TCP connection.

SEs are managed by the SE Issuer by default, but they can also be managed by a Trusted Third Party (TTP) called Trusted Service Manager (TSM). TSM manages all or part of a SE and applications’ lifecycle on behalf of SE Issuers, MNOs, and SPs using OTA infrastructure. Currently most MNOs are capable of providing OTA solutions by using their infrastructure and may even act as TSMs [104,105].

Management of SE can also be performed by multiple TSMs collaboratively by storing configuration data containing management rules and a description of the card content in the SE [105]. In order to manage an application in the SD, TSM requires keys for the SD, which are generally provided by the SE Issuer according to pre-defined business and technical agreements [104].

Controlling access to the SE is yet another important part of SE management. Smartphone applications may face some security vulnerabilities. Use of keyed authentication can be sniffed and storing PIN hash values inside the application would make it vulnerable to attacks that reuse the hash values. Thus, instead of enforcing the SE access control by the smartphone OS, it should be enforced by SE itself [97].

### 3.3. Further Research Opportunities

SE is a significant asset used in card emulation mode applications. SE options have lessened in the recent years by focusing on HCE and UICCs. The open research areas that can be examined, improved, and evaluated by academicians, researchers, and practitioners of SE are indicated below:

- Secure communication protocols for Cloud-based HCE transactions.
- Cloud-based reliable HCE architectures.
- Use cases and prototype implementations of Cloud-based HCE (e.g., access control, ticketing).
- Performance analysis of Cloud-based HCE.
- SE access control models for Smartphone Oss.
- Alternative models for HCE.
- Methods to enhance HCE security.
- Performance and security comparisons of UICC, embedded SE and HCE.
- Connectivity between Cloud-based HCE and Smartphone.
- Cloud based HCE’s off-line running models.

## 4. NFC Applications in Service Domains

NFC technology covers a wide range of applications [106]. In this part, we analyze many NFC technology application domains including healthcare, location, finance, social networking, entertainment, education *etc*. In this section open research areas are clarified separately in each section as well.

### 4.1. Healthcare Applications

The health ecosystem consists of a variety of actors including individuals such as patients, doctors, nurses, and pharmacists; organizations such as hospital administrative management and drug reservoirs; and materials such as drugs and medical devices. NFC can facilitate effective interaction among patients and other stakeholders such as doctors, pharmacies, and even enable the monitoring of drugs. Accurate data exchange between patients and doctors promises proper treatment; accurate data exchange between patients and pharmacies helps efficient and proper drug purchases; accurate data exchange involving patient-drug interaction can manage the risks such as Adverse Drug Event (ADE) and Adverse Drug Reaction (ADR), or overdosing.

There have already been affirmative studies on health applications up to now, but greater effort is still needed to create more satisfactory results. The domain of health can potentially benefit from NFC technology in the following issues:

(a) Healthcare Management Systems.
(b) Diagnosis and Medication by Professionals.
(c) Specific applications for the Disabled, Elderly, and People with Chronic Diseases.
(d) Self-Diagnosis and Medication.

#### 4.1.1. Healthcare Management Systems

The use of NFC in healthcare management systems is reported to some extent in the literature. In one of the studies [107], check-ins at doctors’ offices and hospitals, staff management, at-home diagnostics, fitness performance, emergency connections after injuries, and pharmacy-related operations are used along with NFC technology. In another study [108], a partial management system is presented in which a technical concept for NFC-based medication and related services are presented.

Medical and health cards have also been studied. Some authors offer a model that includes dissemination and usage of health cards during examination in hospitals or other healthcare centers [109]. The paper proposes using either an NFC tag or a smartphone to embed the health card, and also explains usage of both card emulation and peer-to-peer operating modes for use cases. The study also examines advantages, disadvantages and technical details of each such option.

Another study on health cards [110] proposes an infrastructure where patient data is stored on central servers, portable carriers (*i.e.*, smartphones and tablet devices) or electronic health cards (eHC). For the patient appointment, an NFC-based system is proposed [111] and the latter deals with the medication phase as well [112].

#### 4.1.2. Diagnosis and Medication by Professionals

Effective medication is another promising area, where NFC can be used for successful results. A model for clinical data acquisition based on NFC technology is studied in [113]. The authors of [114] examined the use of NFC tags to create information spots, pertaining to a comprehensive healthcare environment and described how nurses can use their smartphones to treat the patient with appropriate medication, medication amount, and timing. Another study [115] discusses the viability of NFC technology and NDEF structure for electrocardiograms (ECGs) and the authors optimize the data format so that low bandwidth capacity of NFC technology can be used efficiently.

In another study [116], an alternative Inpatient Medication System using NFC technology is proposed which aims to guard against adverse medication administration in hospitals. The proposed protocol authenticates doctors and nurses using the system, and matches them with the hospital equipment appropriately. The system aims to decrease the medication errors and identification of the source, place and time of the error are also recorded.

#### 4.1.3. Applications for Disabled, Elderly, and People with Chronic Diseases

NFC technology can also be used for the disabled, the elderly and people with chronic diseases. In [117], an NFC-based model that enables interaction of a blind person with the environment through voice-augmented objects is presented. An NFC tag containing voice-based descriptions is attached to each object within the user’s surroundings, and the user can make use of a smartphone to scan the surrounding augmented objects to verbalize their identity and characteristics. Similarly, another study [118] proposes using NFC technology at bus terminals for the safe movement of visually impaired people. Some researchers also propose expanding the transportation system with NFC technology for visually impaired pedestrians [119].

Assistance systems to help visually-impaired pedestrians already exist in many modern living areas; some of them create unexpected results contradicting the design parameters however. At road intersections where many crosswalks exist, the information from multiple beacons is transmitted and the sounds broadcast from such systems can confuse visually-impaired pedestrians. Some researchers propose a model to prevent this chaos by making use of NFC technology [119].

In a study that focuses on Alzheimer’s Disease [120], an application is proposed that aims to rehabilitate patients by using NFC tags attached to objects such as drawers and doors. In another study [121], the authors also propose a system for Alzheimer’s Disease patients that aims to enhance and stimulate the cognitive abilities of patients. A patient uses a smartphone as well as tangible user interfaces those are equipped with NFC tags to improve the patient’s performance in real-life situations.

A further study consists of a smart poster application that is offered for the elderly and for people with impaired fine motor skills [122]. The proposed application allows patient monitoring, electronic acquisition of well-being data from the patient’s home, and real time representation of patient data.

#### 4.1.4. Self-Diagnosis and Medication

Some studies on self-diagnosis and medication are also performed using NFC. In [123], the authors present an electronic data capture system based on NFC that makes use of smart posters and allows patients to assess their health status. The poster consists of pre-set questions about possible problematic areas towards the diagnosis of illness, which can be answered by the home-care patients using their smartphones. Medication errors that occur during medication administration stage are a serious issue in healthcare. In [124,125], the authors propose a solution that uses NFC technology for nurses to administer the right medication to the right patient in the right dose at the right time and via the right route during the medication administration stage.

In another study [126], a remote monitoring solution was developed to record patient’s medication intake by using NFC-enabled smart medication blisters and a smartphone. Another study [127] also explores self-medication processes and offers a model that implements the lifecycle of prescription preparation, getting the drugs from the drugstore, and applying medication properly based on the directives of the doctor. In another study [128], a patient terminal concept with NFC technology was developed for patients suffering from chronic diseases and their attending physicians. The system was used in a number of proof-of-concept projects with hospitals and health insurance companies and in clinical trials.

Some authors present an approach for ubiquitous/pervasive access to guidance and decision support services for medication safety with an intended audience of patients/citizens and even healthcare professionals [129]. The proposed model helps patients become aware of ADE effects from potential misuse risks such as risky drug-drug interactions or inappropriateness of a specific drug to their current medical, physical, or mental condition. It may also be used as a decision support system for preventing ADE conditions in this manner. In another study [130], the authors present a model so that NFC can be applied in a pharmaceutical system to examine drugs in order to detect cases of ADR.

In a study on dieting [131], a scenario is presented where the dieter uses a smartphone to get data about food nutrition just before consumption, and the application aggregates daily amounts to check the acceptability of food just before consumption, acting like a decision support system.

Researchers have studied the integration of WSNs with NFC in recent years, especially in healthcare monitoring systems. Attaching and embedding sensors to the human body is one of the deployment alternatives. A low-power wearable biosignal monitoring system is studied that can communicate with smartphones to check vital signs such as heart rate [132].

In [133], the authors propose a system where sensors are deployed on the human body. The data from sensors are collected by an intermediate device that uses ZigBee technology and then the data are transferred to the smartphone afterwards with NFC.

It is true that the power consumption of such wearable systems must be reduced and components need to be miniaturized to make them easier to carry [134]. The same paper offers wearable sensors and other health monitoring devices that switch to standby mode in order to reduce power consumption.

Empowering NFC-enabled medical devices also increases the usage of NFC technology in healthcare systems. In a study on NFC-enabled medical devices [135], the authors propose integrating a blood pressure meter and a pedometer on an NFC reader to receive the corresponding data and further transfer them to the database on the server it is connected to.

In [136], two medical wireless sensors—pulse meter and ECG—are attached to the patient’s body for monitoring the activity of the heart, which is further transferred to the patient’s smartphone, and then transferred to hospital’s database when a medical assistant touches her smartphone to patient’s Smartphone. In another study [137], the authors developed an interface that can be attached to an existing sensor or measurement device, and enables monitoring and control of these devices using NFC technology and a cellular data network.

#### 4.1.5. Further Research Opportunities

A significant number of health-related NFC studies have been performed in the literature. Nonetheless, there are still open research areas in the healthcare domain which may potentially integrate NFC technology with health applications:

- A complete healthcare management system by NFC and prototype implementation.
- Access control of doctors and health institutions to patient records on SEs of smartphones.
- Managing medical data (*i.e.*, how and where to store which part of the information).
- Medical data structure and format.
- Medical care of patients at home.
- NFC based healthcare system requirements for elderly and disabled people.
- Re-designing NFC capable medical devices (*i.e.*, a smartphone could easily get the measured data, or the device can get the patient data from the smartphone, *etc*.).
- Cloud HCE based health card applications and prototype implementations.

### 4.2. Location Based Services

Location-based services (LBSs) are key technologies in the mobile sector [138]. In this manner, smartphones are used in diverse environments such as buildings, outdoor areas, vehicles, and even underground settlements. This diversity makes it necessary to integrate different localization technologies to provide sufficiently accurate estimation of location in the majority of vicinities.

Using the geographical position of users’ smartphones enables proposing many LBSs which include services like tracking, navigation, routing, identification, access control, and so on. Several studies have already been performed which combine LBSs and NFC technology.

#### 4.2.1. Tracking Applications

In one study [139], integration of LBSs with NFC technology is performed to track users’ behavior and to improve user experiences. Cities like Frankfurt and Oulu are examples of tagged environments enabling smartphones to retrieve location specific data from available tags [140]. The tags may be attached to park meters, bus stops, street lamps, or some other locations; and may create an infrastructure for users to seek information about the local services. After addressing such examples, the authors of [141] offer the creation of a location-based mobile Wiki. Moreover, real time location data may enhance social networking, where the proposed LocaTag system uses instant messaging tools, location data, and NFC technology.

The authors of [142] analyze the basic requirements for trusted location data and propose a smartphone-based solution, which allows users to assure their current location, and Service Providers to obtain reliable information on users’ location.

In another study [143], an NFC check-in system is presented, which benefits from Implied Location Based Services (ILBS), smartphones, and NFC tags. In order to enable a specific action, the user has to physically be at a specific location; allowing more intimate interaction. Advantages of the implied NFC check-in service with respect to manual check-ins (*i.e.*, Facebook, Foursquare and so on) are presented in the same study.

#### 4.2.2. Navigation and Routing Applications

Other significant NFC-based LBSs are routing, guidance and navigation services. It is known that cellular-based positioning systems do not perform well in indoor environments. There exist valuable NFC-based navigation applications for indoor environments including shopping malls, hospitals, university campuses, museums and so on [144,145,146,147,148].

The authors of [144,145] present an NFC-based navigation system they call NFC Internal, which orients the user by touching a smartphone to NFC tags those are spread inside the building. A prototype implementation for a shopping mall is also performed and performance evaluation of the system is conducted in terms of several parameters, including security, privacy, cost, accuracy, performance and robustness. Another study [146] proposes an NFC/Inertial Navigation System (INS) localization system which reads the NFC tags are previously placed on the floor of the building. The accuracy of using NFC technology for localization in indoor environments is guaranteed due to the fixed positions of the NFC tags [145,147].

#### 4.2.3. Identification and Access Control Applications

Location-based identification and access control services making use of NFC technology are also reported in the literature. An access control system based on two-factor authentication is provided that utilizes a smartphone (*i.e.*, something that the user has) and user’s photo that has embedded hidden password (*i.e.*, something that the user knows) to overcome the disadvantages exhibited by access control systems using access cards [149]. The proposed system combines NFC technology with an information hiding technique, steganography. In another study [150], the authors provide a two-factor access control system for unlocking a door where biometric fingerprint recognition is used for authentication, and NFC is used to transmit the authentication data to the computer controlling the door. In another study [151], an overall identification and utility system for Hajj pilgrims is also described which includes pilgrim identification, prepaid services, lost pilgrims, e-purse and so on.

Another important application area of LBSs is location-based attendance and identification that is especially useful in schools. Innovative NFC identification and attendance services [152,153,154] have been tested and implemented to simplify attendance monitoring and tracking in education. Another study of note presents a NFC-equipped smart classroom to automate attendance management, locate students, and provide real-time feedback [155]. NFC-enabled attendance management systems conserve time and reduce paper work since students use their NFC smartphones to register their attendance automatically.

#### 4.2.4. Further Research Opportunities

The LBS area needs more application-related studies than theoretical ones; hence, solid use cases would be appreciated. There are still some open research areas, which are listed hereunder:

- Integrating wireless sensors with NFC for tracking user behavior and enabling valuable services.
- Identification and access control use cases and prototype implementations in diverse areas.
- Usability studies of LBSs.

### 4.3. Financial Applications

One major motivation for technological developments is obviously their prospective economic impact. For example, research and development efforts on NFC-based payment services always gain remarkable support since payment services immediately affect monetary benefits. Ticketing, coupons, and loyalty are additional financial services in this area.

#### 4.3.1. Payment, E-Money and E-Wallet Applications

In the last decade, Card Issuers (CIs) have introduced contactless credit and debit cards which can be used very intuitively and seamlessly. NFC technology enables smartphones to be used for contactless payment instead of credit or debit cards.

A payment protocol, named as MobiTag [156], enriches the EMV protocol to upgrade it from a payment protocol to a complete transaction protocol. Mobitag enables using, redeeming and acquiring other valuables representing vouchers, coupons, or tickets. The authors of [157] present a system that collects tolls using NFC technology. Another study [158] emphasizes the importance of the agreement between the actors in the NFC ecosystem for enabling beneficial payment services.

Most of the existing payment schemes are either user-centric or institutional-centric; either a person or an institution may purchase goods or services. Some authors [159] propose a model that enables cars to make payments by using pre-paid accounts.

E-wallets are yet another concept for increasing the efficiency of monetary processes. An e-wallet is analogous to traditional wallets which store credit cards, debit cards, gift cards, loyalty cards, and so on. Currently, two popular electronic wallets (e-wallets) exist in the market: Google Wallet [160] and Apple Pay [161].

#### 4.3.2. Ticketing Applications

In parallel to the integration of computers into our life, electronic tickets (e-tickets) have largely replaced the classical form of tickets. With the introduction of NFC into ticketing services, many studies have been realized to create NFC-based ticketing models, prototypes, and projects. An e-ticket prototype that implements NFC technology has been developed [162] and the usability of the prototype is also demonstrated in the same study. Another project [163] made use of NFC technology in e-ticket-related activities. According to the report, a user can purchase tickets, link the tickets to his/her e-wallet, buy items at the food and drinks stands in the hall, and perform similar actions.

In a study [164], a scenario for the integration of an NFC-based e-ticket model into a public transport system is introduced. The model is pretty much flexible, so that the user may travel using any form of public transportation vehicle such as trains, trams, or buses, and may transfer between different kinds of vehicles during the journey. The vehicles may be operated by different companies, and the journey may even include different countries. In another study [165], a prototype implementation of NFC-based ticketing application is presented. Another NFC ticketing system that is provided by OTA provisioning and off-line authentication using public key cryptography is also proposed in [166].

Some authors [167] have proposed an NFC-based e-invoice (electronic invoice) remitting scheme using NFC peer-to-peer communication and discuss how it fulfills major security requirements including authenticity, integrity, and non-repudiation. The proposed system is also implemented and tested in Taiwan’s e-invoicing system. In another study [168], the authors present a solution where a smartphone in reader/writer mode interacts with a ticketing system in card emulation mode.

#### 4.3.3. Coupon Applications

Using coupons is an option for companies to promote their products or services. The customer benefits from coupons by a discount or rebate during redemption, whereas the company benefits from customer loyalty. Electronic coupons (e-coupons) and mobile coupons (m-coupons) have been the subject of many studies. In studies on m-coupons [169,170,171], the authors propose a model for enabling m-coupons, which can be downloaded from a smart poster or a newspaper that contains NFC tags. The user can then redeem the m-coupon at the cashier during checkout.

In a study termed WingBonus [172], an NFC-based model is defined; and its dissemination, distribution, supply, validation, and management of vouchers, loyalty point cards, *etc*. are further explained. WingBonus can also be used as a platform for advertising products and managing market research. The coupons can be managed through both its web site, or by a mobile phone. WingBonus was later extended to include QR codes [173] and is claimed to be fully adaptable to the requirements of marketing campaigns, voucher providers, shop or retailer infrastructures and mobile devices and purchasing habits. The security of the voucher is also studied within the same work as well.

#### 4.3.4. Loyalty Applications

Some authors [174,175,176,177] have proposed NFC Loyal to manage data interaction and sharing among payment and loyalty applications. NFC Loyal initially stores the information provided by the payment applications, and then shares them with the loyalty applications through a secure communication model; hence loyalty firms benefit from these valuable data and provide loyalty services for users. The immediate outcome of NFC Loyal is the increase in purchase frequency of customers; as well as a better business plan among payment firms, loyalty firms, and improved conditions for the user.

#### 4.3.5. Further Research Opportunities

Financial application studies so far are mostly commercial, and only a few academic studies exist in the literature. Security of financial applications has been studied in detail, and we handle those issues in Section 5. The open research areas in terms of financial applications are as follows:

- E-Wallet use cases and implementations including loyalty cards and coupons.
- HCE based financial applications and prototype implementations.
- Usability studies of NFC-based financial applications and their comparison with non-NFC alternatives.

### 4.4. Social Networking Applications

Recently social media applications have been attempting to apply obligatory rules to tether virtual identities to the actual person, so that the declared properties would be real. NFC can be used in that kind of unobjectionable case to bind the real and virtual identities to improve functionality. This phenomenon is studied by taking conference attendance as a case [178]. In this study, each attendee uses a smartphone programmed to expose his/her Social Network Service Identity (SNS ID). The proposed model aims to bind an actual identity to the SNS ID of the participant. Smartphones get in touch with NFC tags and use cellular data network to communicate with the conference database server when some data is to be exchanged. The expected outcome of the study is improved communication between participants, as proposed by the authors. A similar work [179] has been performed as well, where authors propose a participant management system for meetings. The system promotes social networking of participants by binding SNS IDs and NFC tags of participants.

Updating status or profile on social networks using NFC is another studied area. In one study [180], NFCSocial is developed that allows a user to update her position automatically, as well as her current mood manually, by selecting it from the provided list. Another study that presents placing project specific NFC tags to each location, termed ‘Hot in the City’ [89], also allows the users to synchronize their location data on the social network.

An application called All-I-Touch [181] helps users to share a product that they wish to buy with their social network by touching the tag that is placed on the product package. In another interesting study [182], the authors propose an application that aims to create a trusted social network, in which a user builds its own online social space through physical contacts. Users touch their smartphones with another person’s smartphone to initiate a relationship or friendship on a social network. Unlike current social networks, the proposed model integrates real life friendship with the online social space.

NFC technology can also be used to gather information. Crowdsourcing is the process of getting the required knowledge—or in broader terms, a service—from volunteer contributors. Crowdsourcing systems face four key challenges; how to recruit contributors, what they can do, how they combine the contributions, and how to manage abuse [183].

Some authors [184] propose a platform that uses NFC tags on urban infrastructure, allows citizens to make public participatory reports to identify problems, and eventually improves maintenance strategies and urban conservation. The platform makes use of social networks by publishing data to increase participation of citizens.

Another study [185] presents a system that gets the musical preference of people inside a music arena and play mostly voted songs. The system also allows people linking their social network accounts such as Twitter, Facebook and Foursquare to increase the dissemination of social information around all networks.

#### Further Research Opportunities

As summarized, some social networking applications using NFC technology are already developed. However, more studies can be performed when compared to the attention given to social networking in recent decades. The open research areas include:

- Usability of NFC in profile and status updates on social networks.
- Information dissemination using NFC technology.
- Requirements for building NFC-based social network systems.
- Conservation of privacy on social networks.

### 4.5. Entertainment Applications

Smartphones will eventually become an all-in-one device as already discussed in Section 1. We will perform most of the activities that we do today by only using smartphones in the very near future; entertainment is no different. As an example, smartphones can already replace remote television controllers. McDonald’s has launched an NFC campaign in Singapore that turns dining tables into interactive racing tracks [186]. Another game, whack-a-mole, is adapted to smartphones by the authors of [187]. In another interesting game that simulates location-based treasure hunting, tasks are stored in NFC tags, after which they are concealed within a natural setting [188]. Players of the game first find a tag, and must perform the task on the tag. A similar multiplayer ubiquitous strategy game based on NFC is studied in [189]. Still another location-based adventure game is proposed in [190]. In the game, players have to solve quests and find checkpoints in different locations by touching their smartphones to previously placed NFC badges.

Another multiplayer game is also developed in [191] which makes use of NFC peer-to-peer mode. Some authors [121] propose two interactive and collaborative games to be used by people suffering from Alzheimer’s Disease. The games aim to enhance and stimulate the cognitive abilities of these patients. An entertainment case on sharing music experiences is developed in [192] by using NFC tags. Finally, a technically similar case [185] examines how the musical preferences of concert-goes are included on the concert playlist, based on the preference ratings.

#### Further Research Opportunities

Games combining geographic locations and NFC are also widely studied since touching an NFC tag provides assurance of the user’s physical location. The research on games and entertainment areas might be extended to study:

- Pleasure gained by playing NFC enabled games.
- Privacy concerns in peer-to-peer games.
- Technical requirements for NFC-based games.
- Learning systems by NFC-based games.

### 4.6. Education Applications

It is seen from the surveyed studies that NFC is potentially related with education and training. After the development of NFC technology, the existing mobile computing and application development courses in universities were devoted to NFC technology in varying proportions, with entire courses on NFC subsequently developing. Some papers [193,194,195] accordingly comment on education applications of NFC.

Smart school and university environments implemented using NFC technology and NFC- equipped classrooms for course enrollment, attendance and registration control, information gathering and related cases have been developed. In one study [195], an anonymous assessment of exam papers using NFC technology is presented. According to the model, each student has NFC tags, which consist of user identification data such as name and a number. As the student fills in the exam paper, she attaches a tag to the exam sheet, and submits it to the exam supervisor. During grading of exam papers, the evaluating teacher does not see any hint of the identity of the student. In another study [196], authors offered a Smart University project that aims to utilize NFC technology in a university environment. The project consists of smartphones, several NFC tags, NFC readers, and servers for enabling different use cases such as class attendance control and registration fee payment.

NFC technology can be used to improve the capability of students towards learning process for educational content. The Bologna process is recognized as the major motivation for integrating NFC technology into universities [194]. NFC technology is applied to a set of university scenarios for the development of the smart university environment. In another study [11], a learning system is developed that enables educational content to be accessible without time and location dependence. The proposed system integrates NFC and home automation technologies and enhances learning process at home. Students use their smartphone’s NFC capability to display educational content, such as slides and videos, on large TV screens/displays. In another study [197], usage of a ubiquitous NFC-based game in learning is proposed. The game makes use of an open course management system that helps educators create on-line learning communities. Some authors [198] developed an NFC-enabled learning environment that supports Japanese conversation practice. Students interact with the system using their smartphones and download learning materials. Moreover, students are able to share the materials among themselves using NFC peer-to-peer mode. In another study that is conducted for easy access to bibliographic sources [199], a pervasive system is proposed to students for accessing, reading, and reviewing necessary bibliographic sources.

#### Further Research Opportunities

Several NFC based education and teaching models exist that have focused on partial issues. However, a complete technological education solution that also integrates NFC technology is beneficial.

### 4.7. Miscellaneous NFC Applications

Further NFC applications which cannot be included in a specific section above discussed in this section. Dynamic NFC Display is an approach to touch-based mobile interaction between smartphones and NFC-tagged large screens. The Dynamic NFC Display concept is studied in [200,201]. A smart poster model [202] is also studied in which the authors switch the role of NFC tag and reader for the implementation of smart posters.

Sme authors [203] have reviewed applications of NFC technology in the tourism field. NFC-enabled tour guiding [204], NFC services in tourism farm [205], NFC smart tourist cards [206] are some of the substantial applications developed in this domain.

In a study of an NFC-enabled shopping process [207] several different processes were implemented including shopping list management, basket management, coupon redemption and payment. In parallel to the promotion of IoT, the Smart Home concept is making use of current technology to improve residential life. The authors of [208] have designed and developed a smart home environment that makes use of NFC.

NFC-enabled environments for museum visitors [209,210], NFC-based dining experience [211], NFC in industrial manufacturing plants [212], automatically connecting to a Wi-Fi access point using NFC tags [213], a car parking model that utilizes NFC for efficiency in finding the way back to car [214], a complete and innovative smart car parking system which also integrates the valet service electronically [215], NFC-based voting [216] are further implemented applications.

## 5. NFC Security

NFC services are subject to store and manage users’ private and monetary information, so NFC services must be able to provide a secure framework to reassure users and thereby motivate demand. The security of NFC technology can be analyzed in the following domains:

- Security of NFC Tags.
- Security of NFC Readers.
- Security of Secure Elements.
- Security of NFC Communication.

### 5.1. Security of NFC Tags

NFC tags are used in the reader/writer mode of NFC. Two use cases are typical: the smartphone may read data from a previously loaded NFC tag, or the smartphone may write/overwrite to a tag. Read and write permissions of tags are important here; unauthorized read or write functions are unwelcome, of course. Physical security of the tags, unwitting actions weakening the system, as well as threats aiming to damage the system are potential risks of NFC tag security.

The aim of an attacker is potentially find a way to manipulate the data stored on the tag such as overwriting malicious data onto the original one, deleting the content of the tag, or even cloning the tag and impersonating it thereafter. An attacker may inject a worm-URL into the tag, eventually causing the smartphone that reads tag to become infected. Denial of Service (DoS) attack is another risk for the tags [217].

It is possible for an attacker to alter data in an NFC tag and hence create a new malicious tag that leads to malicious content sharing with the attacker [217,218,219]. Using NFC Forum’s Signature Record Type Definition in NDEF messages [80] initially seemed to be a good counter, however, vulnerabilities were later discovered in these record types [84,218]. According to the results of these studies, Signature Record Type Record needs improvement for to create secure NDEF records.

As a prevention mechanism, read and write privileges can be defined for the tags, so that only the authorized users can make use of the service. For checking authorization, authentication mechanisms are required, and there are two main authentication categories that a tag can perform. The first option is off-line authentication, which is performed between the smartphone and the tag. When there is no previously shared secret—such as password or a key—between the smartphone and the tag to build a secure communication, off-line authentication is a challenging process since the tags have a low computational power. In on-line authentication techniques, the smartphone connects to a server that contains a database of secrets. After obtaining a tag’s secret from the server, it ascertains whether it matches with the secret on the tag or not [219,220].

Recent studies have examined authentication and encryption on tags. One of these demonstrated a cryptographic challenge-response protocol is executed between the smartphone and the tag that is based on Public Key Cryptography (PKC) and Public Key Infrastructure (PKI) [221]. The protocol successfully detects illegal modification and cloned tag cases. In another recent study [222], a security-enabled passive NFC tag is designed and implemented which supports authentication using symmetric cryptography. The tag is also able to digitally sign the data using asymmetric cryptography. These recent studies provided authentication and encryption/decryption on tags and eventually overcame the challenges faced in off-line authentication. However, studies on new authentication protocols and analysis of recently developed tags are still required to eliminate obstacles on off-line and on-line authentication of NFC tags.

### 5.2. Security of NFC Readers

NFC readers have the central role in card emulation mode, though they have the potential to act in peer-to-peer mode as well. NFC readers can be wired or wirelessly connected to the backend server. All card emulation mode services process sensitive data such as credit card credentials; hence, the communication between readers and the servers must be secured via encryption. Potential attacks may be realized towards either the NFC reader itself, or towards the communication between NFC reader and smartphone. There are few significant studies in the literature on this topic thus far. Furthermore, since NFC readers are almost identical to RFID readers, NFC readers can be subject to destruction or removal [223].

### 5.3. Security of SEs

SE is an essential part of the NFC card emulation mode as explained above. Security of SE is vastly important, since the whole purpose of creating SEs is to handle sensitive data. Consequently, strong keys are used in encryption to protect SE-related data; and applications would be installed to an SE only after authorization. SDs as part of the SE are also embedded with secret keys to secure the communication with Service Providers.

Since SE security is the crux of all NFC-based financial services; security of SEs and countermeasures are well studied by MNOs, financial institutions, and other SPs. Moreover, GlobalPlatform performs standardization on SE security.

Some studies exist in the literature which analyze SE security, and some of them propose new frameworks. In a study in the literature [224], several possible attack scenarios on the SE are presented and studied. The first attack was DoS attack. After authentication tries and failures on a SD, the card’s state is changed to TERMINATED. As a result of the state change, new applets cannot be installed or existing applets cannot be removed; nevertheless, all pre-installed applets could still operate. It can be argued that, in this case DoS attack was partially successful. The study also indicates that every application can make an authentication attempt to the SD, thus a malicious code can be injected into a smartphone application that performs a DoS attack and easily put an SD into a TERMINATED state. In the same study, a relay attack is performed by installing a malicious application to the user’s smartphone. The communication with the SE is relayed across a network to a card emulator. Using such means, this card emulator performs card emulation with the remote SE.

The applets installed on SEs may also cause vulnerabilities if erroneous code exists; hence prevention mechanisms are required. In one study [225], the authors propose a genetic algorithm that searches for vulnerabilities in SE applets. Another study [226] also examines a SE applet—Google Wallet—for vulnerabilities in software-based relay attacks. The communication between smartphone application and SE applet, as well as the interaction between NFC reader and SE are analyzed. It is found that the applets were not sufficiently protected from the smartphone applications and hence were vulnerable to software-based relay attacks. Moreover, it is seen that the PIN protection which is controlled by smartphone application can be bypassed by sending lock and unlock commands to the applet. The study also notes that Google fixed the software-based relay attack in the next version of the application. In another study [227], the security of tickets stored on SEs is discussed and a new protocol is proposed that performs ticketing process between two entities which do not trust each other. It is seen that at the usage stage, ticket cloning can be prevented by online verification.

Using Personal Identification Numbers (PINs) to secure the SE is a trivial option, and several other biometric techniques including fingerprint, voice recognition and face recognition can be used as well.

An intelligent authentication framework benefiting from various authentication methods is developed in one study [228]. The security framework defines security zones and their minimum requirements, and determines the security level of device, based on user’s previously performed activities. When a transaction is requested, if the corresponding application’s required security level meets the current security level of the device, the transaction is allowed without any authentication. However, if the security level does not meet the criteria, the framework uses an authentication method such as fingerprint, voice recognition, face recognition, password, or PIN. Another study on authentication for access control systems [229] makes use of steganography, cryptography and graphical password to deploy a secure two-factor authentication system. The developed system enables users to use their Smartphone as a key for access control securely.

The security of developed protocols can be further analyzed using security analysis software. A study analyses an existing NFC coupon protocol using the Casper/FDR tool and discovers an attack that enables an intruder to cash a coupon even if she is not allowed to do so [230]. Also, a mutual authentication between the user and the cashier is proposed in the study, which counters the attack.

### 5.4. Security of RF Communication

Since NFC uses wireless communication, a variety of attacks is possible on the communication between two NFC devices. The short range of NFC is an advantage to maintain secure communication, but several other attacks such as eavesdropping, MIM attacks, data corruption, data modification, and data insertion are still possible. As a matter of fact, several countermeasures are already proposed in the literature.

Eavesdropping is possible by using a receiver, suitable antenna, and RF signal decoder [231]. The attacker can use a high capacity powerful antenna and intercept the communication between NFC devices over a greater distance [231,232]. In order to be protected from eavesdropping, a secure communication channel needs to be maintained [231,232,233]. NFC-SEC protocol (NFCIP-1 Security Services and Protocol) is promoted to exchange a secret key between NFC devices for symmetric key encryption [231,234]. To prevent eavesdropping, a scheme is also proposed that leverages the noisy wireless channel to obtain provable secrecy for both card emulation and peer-to-peer modes [235]. The scheme distributes the key on multiple frames in the communication, and the passive eavesdropper becomes completely blind when she misses at least one frame.

Modifying the data in NFC communication may cause Data Corruption. If the attacker converts the data to an unrecognized format, the action may cause a DoS attack since the receiver will not be able to recognize the incoming data and will not be able provide the intended service thereafter. In order to counter data corruption, active NFC devices may check the RF field strength of the communication. Since the attacker needs to produce a higher amount of power than the typical RF field power, the active NFC device will detect this high RF field power caused by the attack [232,233].

To cause a Data Modification attack, the attacker may replace the original data with a valid one that a receiver may understand and accept. In a study [233], it is stated that a data modification attack is feasible for certain bits in modified Miller Amplitude Shift Keying (ASK) and for all bits in Manchester coding with 10% ASK. However, the attacker needs expertise in the radio communication field, and should be able to handle amplitude modulations of the transmission [232]. There are several methods to counter data modification attacks. The first option is to decrease the Baud rate to 106k in active mode; this option potentially makes the communication vulnerable to eavesdropping, however. Another method is to counter the attack by monitoring RF field continuously for such an attack, and cease the communication in case an attack is detected. The third and last method to counter data modification attack is using a secure communication channel [231,232].

In the case of Data Insertion attacks, unwanted or falsified data can be inserted into the actual data transmitted during the communication. The success of a data insertion attack depends on the response time of the target device. If the target device needs a long time to respond, data insertion may be possible. Various methods exist to counter data insertion attacks. The first option is for the target to respond immediately; the second option is continuous monitoring of the channel by the target; and the third option is to use a secure communication channel [231,232].

Since NFC operates in a very short distance, a Man-in-Middle (MIM) Attack is very hard to execute at such a communication distance [231,233]. Even so, using active-passive communication mode is recommended as an additional countermeasure. Moreover, if an active device monitors the RF Field while sending the data, the attacker would be detected easily [231,232]. It is also stated in a study [236] that a relay attack is possible on communication and usage of nonce prevents MIM attacks.

In order to prevent relay attacks, the temperature data of the temperature-enabled tags are used [237]. The surface temperature of the tag is measured both by the tag itself and by the smartphone, and they both use distance-based validation. Both of the measured temperatures should be very close to each other, and verifying the physical proximity of tags by using measured temperatures is used to prevent relay attacks.

Most attacks in RF communication can be prevented by establishing a secure channel between NFC devices. Diffie Hellman-based key exchange protocol or Ecliptic Curve Cryptography (ECC) can be used to establish a secret key; and the generated key then can further be used in a symmetric encryption protocol such as 3DES [233] to enable channel security.

Authentication protocols are one of the most studied issues in NFC communication security. One study has developed a Needham-Schroeder-based secure mutual authentication protocol which aims to guarantee authentication and confidentiality between SE and NFC readers in card emulation mode [238]. The protocol enables devices to share a session key, which will be used for secure transactions thereafter. It uses a trusted entity termed as Authentication Server, which verifies reliability of entities, authentication of SE to NFC reader, and authentication of NFC reader to SE. Another mutual authentication protocol for reader/writer mode communication is proposed in [239]. The protocol uses ECC, and provides mutual authentication and a secure environment for demanding transactions. An additional mutual authentication protocol that is designed for mobile payment is also presented [240], which deals with card cloning, skimming, downgrading terminal and relay attacks. The protocol mutually authenticates the customer, smartphone, NFC reader, and bank before the transaction; and uses one time password (OTP) to generate session keys for encryption purposes. The study also analyses the protocol using Casper security analysis software. Some authors [236] propose a high-speed processing of authentication and key agreement for NFC payment, and also present a method to perform secure communication. In a further study, two authentication protocols that also ensure mutual authentication between two devices are proposed [241].

One-way authentication protocols are also studied in the literature. A lightweight ECC based authentication protocol taking into account resource constraints of system such as smart cards is submitted [242]. A new mobile payment protocol, EMV-TLS, based on modified EMV chip has also been developed [243]. The protocol solves the issue of trustworthy remote use of a chip by using an NFC smartphone that currently has an Internet connection.

**Table 1:** Vulnerabilities, attacks and countermeasures.

	Vulnerabilities and Attacks	Countermeasures
**NFC Tags**	Tag Manipulation (*i.e.*, NFC Worms, Phishing, DoS Attacks)	Digitally Signing Tags Using Tag Authentication
	Tag Cloning and Tag Impersonation	Digitally Signing Tags
	Tag Replacement and Tag Hiding	Protecting Tags with a Physical Shield
**RF Communication**	Eavesdropping	Using Secure Communication Channel
	MIM Attack	Attack is nearly impossible Using active-passive communication mode (RF field is continuously generated by one of the valid parties) Listening RF field when sending data
	Relay Attack	Using nonce Using physical proximity based information (e.g., temperature)
	Data Corruption	Checking the power of RF Field
	Data Modification	Changing Baud rate Monitoring RF Field Using Secure Communication Channel
	Data Insertion	Response with no delay from answering device Listening the channel by answering device Using Secure Communication Channel
**SE**	DoS Attack	Solutions needed
	Relay attack	Disabling internal mode communication
	By passing the applet PINs those controlled by Smartphone applications	Two-factor authentications New solutions needed
	Vulnerabilities in applets caused by erroneous codes	Genetic algorithms to search for vulnerabilities in applets Formal security analyses
	Cloning an asset (e.g., ticket, coupon)	Online verification before usage

Privacy is an important topic in NFC security, however, there are not many studies on preserving user privacy in NFC communication. In one of the few studies on this subject, protection methods are proposed for users to protect their privacy. The proposed methods try to hide the user’s identity, but the user’s identity can still be confirmed by the TSM whenever required [231]. Another study [244] investigates privacy issues of NFC payment services; and a specific framework is designed and applied to perform the analysis. In another study [245], an anonymous car rental protocol is proposed where a TTP knows the users’ real identity, however car hiring SP and malicious users are unable to break users’ anonymity. The developed protocol also counters replay and MIM attacks; and provides anonymity, confidentiality and secrecy at the same time. We provide in Table 1 a brief summary of all possible security vulnerabilities and attacks of NFC-based systems and the corresponding countermeasures.

### 5.5. Further Research Opportunities

From the technical point of view, some security issues in NFC technology are already solved and standardization is mostly provided as well. However, there are still unsolved security issues. Related with analyzed studies, the following open research topics are identified to be a helpful guide for the academicians and researchers with an interest in NFC security issues:

- Studies on new authentication protocols and analysis of recently developed tags for eliminating obstacles on off-line and on-line authentication of NFC tags.
- Creating new protocols/mechanisms on off-line and on-line authentication of NFC tags.
- Analyzing security of off-line and on-line authentication of NFC tags.
- Development of security mechanisms for protecting NDEF records within a tag.
- Formal security analysis of proposed applications.
- Experimental security analysis of HCE.
- Security analysis of protocols that makes use Cloud-based HCE.
- Preventing DoS attacks on SEs.
- Experimental comparison of SE alternatives in terms of vulnerability.
- Mechanisms to prevent relay attacks on SEs.
- Mechanisms for preventing the PIN bypass of SE applets that are controlled by smartphone applications.
- Proposal of NFC specific alternative key exchange protocols to prevent various attacks on RF communication.
- Mechanisms to provide user’s privacy.
- Exploration of various NFC service domains in terms of privacy, sensitivity and ethical issues.

## 6. Usability

As science and technology improve, some options explode, while others vanish. The lifetime of new technologies does not merely prove or disprove the technological superiority of the exploded models, and *vice versa*. There are some additional parameters, which affect success of the emerging technologies. One major parameter that determines the survivability of the emerging technology is its acceptance by users. Usability, in this sense, is the ease of use and learnability of the methodology it offers; in this sense NFC technology is not privileged at all.

The communication paradigm between a smart object and the smartphone is known as a mobile interaction technique, and the currently available techniques are Touching, Pointing, and Scanning [246]. By using the Pointing interaction technique, the user can select a smart object by pointing it with the mobile device. Scanning interactions allow the user to get a list of nearby smart objects by using a wireless mechanism, and then select one of them afterwards. Touching is also NFC’s communication paradigm, and occurs when a mobile device is touched to a nearby smart object. Touching is a natural and possibly the simplest interaction technique, since all that a user needs to do is to touch the mobile device to the smart object.

Some usability studies on the Touching paradigm have already been performed in the literature. One study presents a usability analysis of the interaction techniques in general; 134 people participated in the test, though the results are not encouraging for a Touching paradigm [246], as based on the given responses, only 25% of the volunteers enjoyed using it. When they were asked to select one of the provided interaction alternatives, a paltry 13% of the attendees selected Touching as their preferred option. In a usability study [247], the user experience of mobile interactions with the dynamic NFC display is investigated in both single user and multi-user contexts. The study with single user context showed the high appreciation of the interaction. In case of multi-user context, interactions were also appreciated, however users seem to prefer single user interactions to multi-user options.

Several interaction methods are discussed by some authors and NFC-based touch-driven interaction is suggested [248]. According to the authors, touch-driven interaction provides users with full controllability, fine-grain accuracy and high usability. Another work [9] points out the advantages of touch-based interaction in terms of controllability and accuracy. It considers NFC touching as a differentiator between RFID and NFC. With traditional RFID technology, the server controls the process, because the RFID antennas are placed all over the environment, and they detect RFID tags as the user navigates around them. On the contrary, in NFC, users decide when to activate NFC interaction themselves. Additionally, behavior monitoring and extracting by sensors is inaccurate, intrusive, and difficult to control. The authors also argue that modeling user behavior using explicit user action—such as touching devices or putting smartphones together—achieves better accuracy and gives user full control of the system.

### 6.1. Overall Usability of NFC Services

For usability analysis, several analysis models such as Unified Theory of Acceptance and Use of Technology (UTAUT), Strengths, Weaknesses, Opportunities, and Threats (SWOT), Analytical Hierarchy Process (AHP), and Technology Acceptance Model (TAM) have been used in the literature.

The UTAUT model is used for user acceptance analysis of NFC in one study [249]. As stated in the paper, the main purpose of the study was to explore the key factors that influence the Behavioral Intention to use smartphone-based services. The authors gathered 189 valid responses from respondent of various genders, ages, education levels, professional occupations, and prior experience with NFC. The results indicate that Effort Expectancy (the degree of ease that is associated with the use of the system) has a positive effect on Performance Expectancy (the degree to which an individual believes that using the system will help her to attain gains in job performance). Also, Performance Expectancy and Social Influence (the degree to which an individual perceives the importance of others’ belief to use the new system) have a positive effect on Attitude Toward Use of Technology, but Anxiety has a negative effect on it. Finally, Attitude Toward Use of Technology is more significant than Facilitating Conditions (the degree to which an individual believes that sufficient organizational and technical infrastructure exists to support use of the system) in affecting Behavioral Intention.

SWOT analysis is a model to critique strengths and weaknesses of a system. In a study [250], telecommunication professionals with prior knowledge about NFC filled out 23 questionnaires. The findings indicate that the positive factors (strengths and opportunities) associated with NFC are potentially more important than the negative factors (weaknesses and threats). According to the authors, this indicates the potential benefits of NFC-based projects.

Another study analyses a rather detailed technical and user feedback in order to compare paper- based and NFC-based tickets [163]. The study searches for answers to the questions such as: is an electronic ticketing system that much faster than a paper-based system; will customers agree with the vision that a single compact card is easier to use than many regular tickets; will event organizers be convinced by a system that provides protection against reselling vouchers, but does not provide a physical proof of vouchers exchanging hands at the time, *etc*. The study performs the tests based on TAM, which models how affirmatively users accept a new technology. The model suggests that when users encounter a new technology, two major factors influence their decision: perceived usefulness (PU) which presents provided performance increase by the new technology; and perceived ease-of-use (PEOU) which presents ease of use of the new technology. The same study shows that when PU is strongly lower than PEOU, the behavioral intention to use NFC is low as well. Technical study showed that using NFC ticketing requires slightly more time than the traditional paper-based alternative.

NFC-based electronic voting is an alternative to the traditional version, but no satisfactory improvement in this topic has been implemented yet. A study performed on voting [216] created a prototype for using NFC in voting and obtained the feedback of 50 voters towards the usage of the new technology in the voting process. The voters also compared the usability of web-based voting with NFC voting and according to the results NFC voting affords insignificantly better usability.

The results of a usability study show that user experiences on the NFC service were moderately positive, though there were mixed results considering the performance effects of NFC service usage [251]. The potential usefulness of proposed future NFC service functionalities was considered quite high.

In another study [252], user preferences between audio-haptic and visual-haptic alternatives were analyzed. In the study, users tap their smartphones to the NFC tags on the products in the grocery store, and the application on the smartphone creates audio and visual alerts to the user if the food contains a high enough amount of sugar to threaten the user’s health condition; the threshold is based on the user’s diet. The study showed that the participants preferred haptic-visual feedback, which was faster than the audio-haptic feedback, whose long vibration patterns were less suitable as an indication of the amount of sugar in products than the visual representation of the haptic-visual feedback. The participants rated haptic-visual feedback as more helpful, easier to learn, and more pleasant to use. These quantitative results were also confirmed by the qualitative results of the questionnaire. Some results also indicated that purely haptic feedback with various patterns (e.g., vibration intensity) could provide an added value, e.g., for visually impaired users.

A pilot test of an application that preserves anonymity of the students during exam paper evaluation by the instructors, was conducted with 28 students [195]. After all students had turned in the exam papers, they were asked to fill out a satisfaction survey. The survey consisted of 12 questions on the privacy feeling by using the application and the process; and the satisfaction rate was realized as 4.22/5. The authors then concluded that students wished to maintain their anonymity during the exam process, and NFC is a leading candidate to ensure this.

### 6.2. Usability of NFC Based Financial Services

The rapid evolution of mobile technologies and the increasing diffusion of smartphones have provided significant opportunities for innovative companies to offer NFC-based payment solutions and value-added services to their customers.

One of the earlier studies that investigated the acceptance of NFC by European retailers was conducted between October 2007 and April 2008 [253]. According to the questionnaire results, the usage of NFC payment was more promising than other options such as NFC Loyalty, NFC Smart Posters, or NFC Coupons. The responders agreed that NFC-based payment had the potential to speed up the payment part of the check-out process at the cashier.

NFC payment is a significant opportunity to turn smartphones into digital wallets. Some authors [254] provide guidelines for the adoption of NFC-based mobile payments, by proposing a research framework to provide a profound understanding of factors facilitating or impeding this. The study focuses on:

(a) product-related factors (perceived usefulness, perceived ease of use, compatibility, perceived security and privacy risk, perceived cost of use, trialability, and additional value of NFC mobile payment);
(b) personal-related factors (personal innovativeness in new technologies, absorptive capacity);
(c) trust based factors; and
(d) attractiveness of alternatives. The authors made use of five academic and 10 practitioner customers. The authors conclude that:
  - Customers are unlikely to adopt NFC mobile payments unless Service Providers shed some light on the outstanding characteristics and differentiation of NFC-based mobile payment.
  - It is beneficial for the entrepreneurs and Service Providers to classify the market into different segmentations, customize, promote and offer services to suit the specific needs of consumers.

The performance and security issues of NFC-based payment solutions with the classical ones have been compared in one study [255]. The paper concludes by claiming that traditional models are more secure, whilst NFC-based solutions are more functional. Another effort [256] performs a study with 1001 users and 13 professionals, working for various NFC payment actor organizations. The conclusions of the study states that the users emphasized the importance of security; the users prefer to use PIN codes to feel secure; and they prefer double-tap (once to learn the payment amount, and then to approve the payment).

Another study [257] analyzed acceptance of NFC payments in detail. The authors applied a questionnaire to 262 respondents with varying demographic properties with respect to sex, age, educational level, professional area, and credit card usage. The study examined the preference of NFC-based payment by using TAM models. The authors conclude that there is a significant and direct relationship between both PEOU and PU on Intention to Use (IU) while Trust (TR) and Personal Innovativeness in Information Technology (PIIT) have significant indirect effects on IU.

In an additional study [156], the MobiTag protocol containing both customer and merchant interfaces is presented, and user acceptance tests are performed as well. The authors collected 34 answers: nine from participants in the laboratory pilot, eight from merchants, and 17 from customers that participated in the experimental pilot tests. The major identified problem was the performance of NFC connection, which made the users find tapping their smartphones slow and complicated, Transactions are sometimes not completed and must be reinitiated. The pilot users performed 10 different tasks and evaluated the tasks as easy to perform. The attitude of customers and merchants were generally very positive regarding the usage of NFC. Authors asserted that the people are generally open to new possibilities offered by mobile payments such as NFC payment and are willing to experiment with it.

Finally, the design, usability, and user experience of user interactions on dynamic NFC-enabled displays have been investigated [248]. The authors conducted two complementary user studies with the prototypes they created. The first study was carried out with single users to assess the overall performance, usability, and user acceptance of the interaction. The second study was carried out with groups of users to evaluate their behavior during the interaction with NFC-based interactive surfaces together with other users. The responders consisted of 11 students and researchers. The users reported satisfaction with the usage of the system overall, and found it easy to use.

### 6.3. Usability of NFC Based Healthcare Services

There is a variety of actors in health-related services including individuals such as patients, doctors, nurses, and pharmacists, organizations such as hospital administrative management and drug reservoirs, and materials such as drugs. The authors of one study developed an NFC-based system to help nursing students to perform patient care tasks with simple interactions, including medication administration, clinical tests, and vital signs supervision, among others [124]. The authors also evaluated the system in two nursing schools. Sixty-two nurses were engaged in the tests and the results assert that about two-third of the attendees found the NFC-based system useful enough.

In another study [127], an NFC-based system was developed to enable self-reporting of instant health status of the user, to represent and analyze real time patient data and to allow direct medical intervention by physicians. The results of a field test indicated that NFC usage is almost as simple as filling out a paper-based questionnaire, as indicated by the authors. Further, NFC technology was perceived as very intuitive and the information quality of each patient’s health status could be improved using NFC technology.

Usability of NFC technology for the elderly, disabled, and immature individuals is rather important. One study [258] consisted of a field experiment which used NFC smartphones as a user interface element so as to enable home-dwelling elderly people to choose their meals to be delivered by means of a home care service. The authors focused on examining the suitability of a touch-based user interface in the everyday life activities of elderly users. The eight-week experiment showed that the touch-based user interface was easy to learn and adopt and the users were able to successfully use it regardless of their physical or cognitive weaknesses.

### 6.4. Further Research Opportunities

NFC technology is generally thought as easy to use and in parallel, and the results of most of the usability studies indicate the same result. However, we also need to mention that users generally experience a wow effect at first time of NFC usage, but when the time passes this passionate desire to use the technology decreases. Moreover, many usability studies concentrate on the usability of the applications with usage of the NFC technology a secondary focus at best. Relating to the reviewed studies, the following open research areas on NFC usability issues are identified as a guide for the researchers:

- Usability analysis of NFC interaction in various service domains.
- Comparisons of the usability of NFC operating modes.
- Analysis of the NFC wow effect.

## 7. NFC Ecosystem and Business Models

NFC technology is made up of several components, which makes it part of a large business environment, cutting across boundaries of many organizations from diverse business sectors. Its large value-chain includes several industries and organizations such as MNOs, banking and payment services, device manufacturers, software developers, other supplementary merchants including transport operators and retailers [8,18]. All stakeholders in the NFC ecosystem have already experienced and agreed on the fact that NFC services to end users cannot be provided by a dominant single actor; so collaboration of the participants is vital.

The standardization of NFC technology is already achieved and valuable developments have occurred, creating a considerable increase in commercially available NFC-enabled smartphones. According to a forecast by IHS Technology, NFC will be included in 64% of the smartphones shipped in 2018, up from 18.2% in 2013 [259]. Moreover, researchers predict that global shipments of NFC-enabled smartphones in 2018 will be four times higher than in 2013 which means most of the smartphone manufacturers have begun to adopt NFC technology in their products as a *de facto* standard [259].

As also mentioned in [260], the business cases and models in NFC ecosystem are still unclear due to lack of common understanding and vision in NFC technology among participating organizations and industries. It is expected that companies shall cooperate with each other to create the added value first, and compete with each other to take the biggest share of it afterwards [261]. A mutually beneficial business model in the financial services could not have been formed yet.

A study examining the deficiencies of NFC services collaborating with existing contactless and smart card standards in a comprehensive approach and categorizes the problems in two aspects: technology and business related [262]. In terms of technology-related problems, some important issues to be considered are:

- Although NFC technology warrants the separation of various applications on the same SE with a high security and minimal risk of interference, certain security specifications prohibit this coexistence. Thus, the management of multiple applications on the same chip is also an unresolved issue.
- OTA service provisioning is a great benefit of mobile technology, which is used to manage SEs without being physically connected to it [263]. However, diverse technical OTA solutions exist with different capabilities that are not interoperable with each other.
- Currently, different SE alternatives are available in the market, hence different NFC service options exist; each actor proposes a different business model that brings more advantage to that actor than others. For example MNO’s propose SIM-based models, since they can control these cards and hence can receive more profit if this model is used, whereas a mobile handset manufacturer provides embedded hardware-based SE models and NFC services to gain more control in the NFC business environment.

In terms of business and managerial problems, the revenue to be shared is enormous; this creates a failure in common understanding and vision in working with suitable business models in the NFC ecosystem. Furthermore, each participating ecosystem organization is powerful in its own sector; causing it to become arrogant and expect the other parties to respect its subjective demands [262].

To enable a beneficial business model and manage Business-to-Business (B2B) relations efficiently, some profitable models and proposals such as User Centric Smart Card Ownership Model (UCOM) [261], Consumer Oriented Trusted Service Manager (CO-TSM) as a TSM deployment model [264], StoLPan’s Host Application Model as a transparent and uniform platform for managing multiple services [265], Platform Manager Model [260], and Role Based Ecosystem Model [266] are already provided.

In all proposed models so far, the main goal is to achieve a successful business model. It is important to drive cooperation of the partners in the ecosystem and also to enable customer acceptance. Currently there is a tremendous amount of work on organizing the contributions and interests of all entities, and better governance of the overall ecosystem.

From the technical point of view, to create sustainable business models for NFC services, some issues need to be solved completely from all involved parties’ perspective. Secure elements play an important role in defining the business model and ecosystem. In the case of SE elements (e.g., UICC, embedded hardware), three components, namely the SE and SE issuer, SE Platform Manager and OTA Provider determine and structure the business model for an NFC service.

As a matter of fact, practical implementation of such proposed SE-based NFC applications and ecosystem related studies are mostly missing in the literature. Some literature reviews [267,268] also indicate that only 9.46% of the NFC literature focuses on NFC ecosystem and business issues. There is a clear need for rigorous NFC research papers to provide a high level of research [268]. Business models of NFC technology need to be clearly considered with methodologies and design principles that have theoretical proof.

HCE, as a new approach in SE, is an important advance in NFC technology which is referred as a “game changer” in the NFC ecosystem [269]. HCE-based NFC solutions completely eliminate the need for an SE Issuer, TSM and MNO to enable NFC services. On the contrary, HCE Solution Provider (HSP) and Token Service Provider (TSP) as new actors, engage with the ecosystem depending on the HCE solution alternative which can be either cloud-based HCE solutions or tokenization-based HCE solutions [269]. We assume that HCE-based business models will create considerable changes in the NFC ecosystem in the near future.

### Further Research Opportunities

There are still some challenging research areas related with the NFC ecosystem. The following open research topics may be of interest to academicians and researchers:

- Development of sustainable ecosystem model alternatives for NFC services including revenue-cost analysis, SE usage, competency and feasibility analysis.
- Examination of proposed NFC applications’ business impacts and models based on various theoretical frameworks.
- Exploration of HCE-based NFC services business models.
- Analysis of business and economic performance of complex NFC applications such as NFC payment, ticketing and transportation.
- Exploration of business case opportunities based on demography, regulation, market structure, and infrastructure readiness *etc*.
- Identification of liability issues, customer care, and division of other related roles and responsibilities between key stakeholders.

## 8. Conclusions

NFC is one major emerging technology of the last decade. Even though it remains a comparatively newborn technology, NFC has become an attractive research area for many researchers and practitioners due to its exponential growth and its promising applications and related services. In this survey, we have covered all aspects of NFC and put special stress on the academic and innovative issues. We have provided a comprehensive, up-to-date review of NFC technology including academic studies as well as some valuable white papers of industry pioneers within the NFC ecosystem. We believe that this survey study will provide a beneficial source to understand the current status of NFC research. There is a clear need for more rigorous publications to address the issues that have been highlighted in the research opportunities sections. Academicians and researchers should focus on these recommended research issues, perform high quality research and disseminate their findings in order to maintain the advancement of knowledge in NFC research and to identify the gaps between theory and practice.
